# A Cross-sectional Comparison of Cleft Lip Severity in 3 Regional Populations

**Published:** 2012-02-03

**Authors:** Alexis Caitlin Lanteri, Bertrand William Parcells, Ana Karina Lizarraga, William Magee, Luis Bermudez

**Affiliations:** Operation Smile, Inc, Norfolk, VA

## Abstract

**Objective:** The purpose of this cross-sectional study was to compare the severity of unilateral cleft lips in populations of Asia, Sub-Saharan Africa, and Northern Africa and the Middle East. We hypothesize that severity of unilateral cleft lips shows significant variation between these populations. **Methods:** Medical photographs of 780 patients with primary unilateral cleft lips treated by Operation Smile during November 2007 were reviewed. Photographs of 352 patients from Asia (China, Philippines, Vietnam, Laos, and Cambodia), 112 patients from the Middle East and North Africa (Jordan, Egypt, and Morocco), and 316 patients from Sub-Saharan Africa (Ethiopia, Kenya, and Madagascar) were analyzed. The severity of cleft lips was determined using the Fisher method, which measures the columellar angle as a deviation of the columella from its normal vertical position. The angle was measured using a protractor with its base positioned along a line joining the lateral canthi. An analysis of variance calculated statistical differences between each region and their respective countries. **Results:** The Asian region was found to have the greatest severity of unilateral cleft lip deformity (*P* < .05). Analysis-of-variance tests show a significant difference between Asia and other regions studied. When stratifying the data by country, the Philippines and Vietnam showed the highest severity. **Conclusions:** The results suggest a heterogeneous pattern of global severity. Unilateral cleft lips with the highest severity were predominant in the Asian region. The observed phenotypical differences can be used in future studies of gene variability or environmental factors to determine the cause of this significant disparity.

The incidence of primary unilateral cleft lip deformity, the most common craniofacial congenital anomaly treated by plastic surgeons, varies widely across the world. While 1 of 800 children in the United States is born with a cleft lip or palate, developing countries demonstrate a higher rate estimating 1 in every 500 to 600 births.^1^ In terms of ethnicity, cleft lips occur in the African American population approximately 1 in 2000 live births, 1 in 10000 live births in the white population, and 1 in 500 live births in the Asian population.^2^ Although the incidence of cleft lip deformity across continents is well documented, there are currently no published data comparing the severity of cleft lips on a global scale.

Understanding this distribution among populations is critical to fully characterizing the social and fiscal implications of a cleft lip deformity. The purpose of this cross-sectional study was to objectively measure the severity of presurgical unilateral cleft lip nasal deformities in regions across the world and stratify which populations are most affected. This study used a method developed by Fisher et al,^3^ which applied objective anthropometric measurements to characterize the severity of cleft lips (Fig 1).

The unilateral cleft lip deformity produces a degree of craniofacial asymmetry that correlates with the cleft severity.^3^ Fisher et al^3^ demonstrated that quantitative measurements of this asymmetry accurately reflected the cleft lip severity as determined by expert plastic surgeons. We used this method of assessment to examine hundreds of photographs taken during the World Journey of Smiles (WJS), a medical charity event hosted by the nonprofit organization Operation Smile during November 7 to 16, 2007.

The results from this study are intended to offer insight into the implications of cleft lips in regions most afflicted by this congenital deformity. We hope that an improved understanding about the severity of this deformity will aid in focusing available resources to areas that require more complex procedures and have higher risk of complications. Furthermore, the results of this study may provide a foundation for future studies to continue examining potential risk factors for these facial deformities.

## PATIENTS AND METHODS

### Patient selection

**Figure 2:**
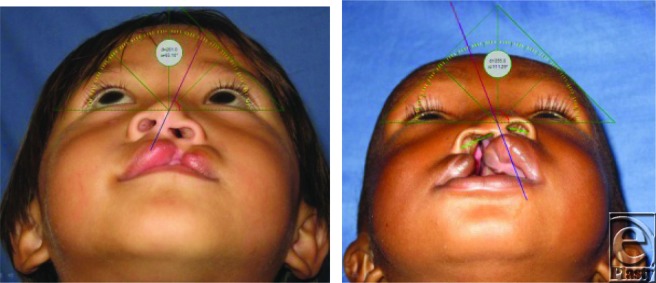
Anthropometric measurement using screen protractor. Photographs property of Operation Smile, Inc. Used with permission.

This cross-sectional study retrospectively examined 760 unilateral cleft lips in multiple regions in the world. All study subjects were drawn from the Operation Smile photograph database. The photographs were obtained during the WJS, a coordinated medical effort that provided surgical repair of 4149 facial deformities in 25 countries over the 10-day period of November 7 to 16, 2007. The study examined photographs from subjects who opted to sign the internal review board–approved consent form (Fig 2).

**Figure 3:**
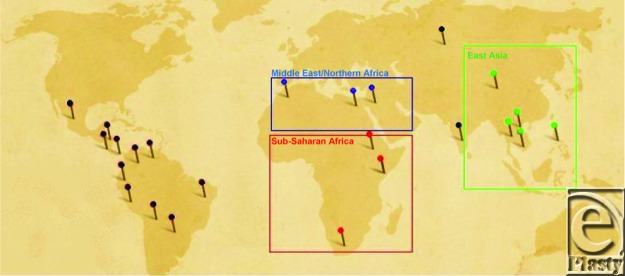
World Journey of Smiles map, property of Operation Smile, Inc. Used with permission.

Subjects were photographed from a standard basal view during presurgical screening and while under anesthesia immediately before cheiloplasty. Photographs were taken by trained patient imaging technicians using a Nikon Coolpix 950 digital camera in macro mode (resolution, 1200 × 1600 pixels). The photograph database organized subjects by site of surgery and then grouped subjects by region. Of the 25 countries involved with the WJS, this study analyzed subjects from the 11 countries in the Asian, the Middle East-North African, and Sub-Sahara African regions (Fig 3).

Subjects included in the study had unilateral cleft lip (with or without a cleft palate) deformity but were excluded if they presented with other comorbid craniofacial deformities. Subjects were also excluded if their photographs did not conform to the standard basal view or if the columella or lateral canthi were obstructed, thus preventing accurate measurement. The study included subjects of all ages and did not attempt to balance a male to female ratio between regions.

In the Asia region, a total of 351 subjects were analyzed from the countries of China (34 subjects), Philippines (144 subjects), Vietnam (94 subjects), Laos (45 subjects), and Cambodia (35 subjects). In the Middle East and North Africa region, 112 subjects were analyzed from the countries of Jordan (17 subjects), Egypt (39 subjects), and Morocco (56 subjects). In the Sub-Saharan African region, 297 subjects were analyzed from the countries of Ethiopia (166 subjects), Kenya (81 subjects), and Madagascar (69 subjects).

## ANGLE MEASUREMENT

A persistent septal-columellar nose deformity of varying severity accompanies a unilateral cleft lip deformity. The base of the columella is pulled to the noncleft side and the nose tip deviates toward the cleft side, thus causing columellar deviation, septal deformity, and anterior nasal spine hypertrophy.^4^ While many prior studies have suggested methods to assess the severity of cleft lips, our study utilized Fisher's assessment via the columellar angle. The method recognizes that unilateral cleft lip deformities produce a degree of craniofacial asymmetry and found that the degree of columellar displacement accurately reflects the asymmetry severity. The columellar angle, measured as the degree of columellar deviation from its normal vertical position, thus provides an objective measurement of the cleft lip severity. Fisher et al^3^ demonstrate a strong correlation between this objective anthropometric measurement and the subjective assessment of an expert panel in the field of craniofacial deformities.

The columellar angle was measured using a protractor with its base positioned along a line joining the lateral canthi. The angle between the vertical axis of the protractor (90°) and a line drawn through the columella was recorded to the nearest 5°. This angle of deviation was recorded for each patient as the “columellar angle.”

## ANALYSIS

The subjects were grouped by both country and region. The mean columellar angle and standard deviation were calculated for each group. One-way analysis of variance was utilized to determine statistical differences between countries and regions, while *t* test analysis was used to directly compare 2 countries or 2 regions. The subjects were also compared by sex and by the side of the cleft.

## RESULTS

Our study examined 781 subjects and the overall columellar angle ranged between 5° and 75° (mean = 34.17; SD = 14.82). The study population demonstrated a significantly greater number of left-sided clefts compared with right-sided clefts (569 left sided vs 212 right sided; left/right ratio, 2.6). There were 492 males and 289 females (male to female ratio, 1.7). The mean columellar angle in females was 35.95°, compared with 32.81° in males, which was statistically significant (*P* < .003).

**Figure 4:**
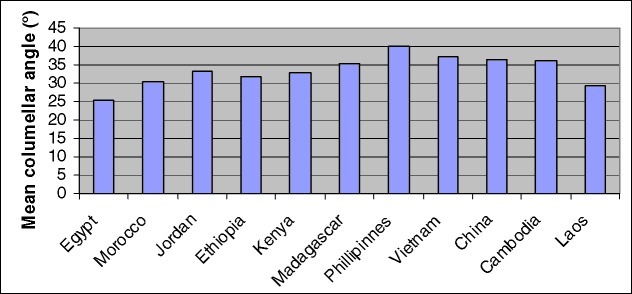
Mean columellar angle as grouped by country. The Philippines had the most severe columellar angle deviation, while Egypt had the least severe columellar angle deviation.

There were 352 subjects analyzed in the 5 countries of the Asia region. The Philippines contributed the largest number of subjects, and its average columellar angle was the largest of any country in the study at 40.00°. However, there was not a statistically significant difference between the mean angle of the Philippines and that of Vietnam, which had the second largest angle (*P* > .13). In contrast, Laos demonstrated the smallest angle of any country in the Asia region at 29.11° (Fig 4). When comparing the columellar angle of countries within the Asia region, there was not a significant difference between the Philippines, Vietnam, China, and Cambodia (*P* > .27). However, when additionally including Laos, which had a significantly lower average columellar angle, there was a statistical difference between the countries of the Asia region (*P* < .01). Overall, the mean angle for the Asia region was 37.06°.

There were 316 subjects analyzed in the 3 countries of the Sub-Saharan Africa region. Ethiopia contributed the largest number of subjects (n = 166), then Kenya (n = 81), and lastly Madagascar (n = 69). Madagascar had the largest average columellar angle at 35.43°, while Ethiopia had the smallest average angle at 31.66°. When comparing the columellar angle of countries within the Sub-Saharan Africa region, there was not a significant difference between countries (*P* > .17). The overall mean angle in Sub-Sahara Africa region was 32.75°.

There were 112 subjects analyzed in the 3 countries of the Middle East/North Africa region. Morocco contributed the largest number of subjects (n = 56), then Egypt (n = 39), and then Jordan (n = 17). Jordan had the largest columellar angle at 33.24°, while Egypt had the smallest angle at 25.38°, which was also the smallest average angle of any country. The overall mean angle in the Middle East/North Africa region was 28.97°.

**Figure 5:**
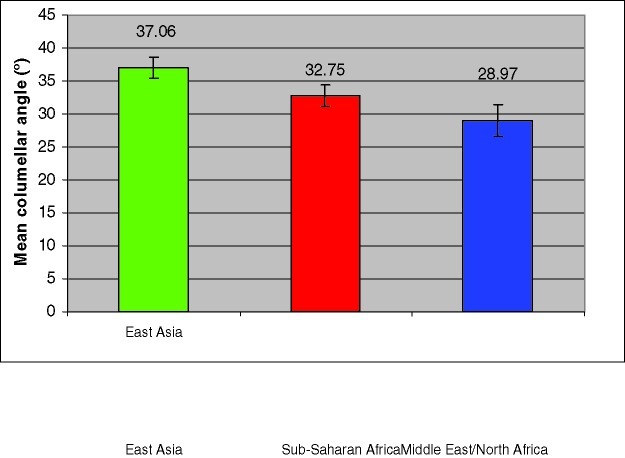
Mean columellar angle as grouped by region. The East Asia mean columellar angle (37.06°) was significantly more severe than those of Sub-Sahara Africa and the Middle East/Northern Africa (*P* < 0.05).

There was a significant overall difference in mean columellar angle between the 3 regions of Asia, Sub-Saharan Africa, and the Middle East/North Africa (*P* < .001). There was also a significant difference between both the Asia region and the Sub-Saharan Africa region (*P* < .001), and the Asian region and the Middle East/North Africa region (*P* < .001). In addition, there was a significant difference between the Sub-Saharan Africa region and the Middle East/North Africa region (*P* < .01; Fig 5).

## DISCUSSION

Epidemiologic studies examining the incidence of CL/P have shown a wide variation between different ethnicities and regions worldwide. Our study appears to be the first to consider such regional disparities in regard to the severity of CL/P. Our study identified trends similar to those of CL/P incidence, whereby the countries with high incidence rates also demonstrated the most severe CL/P deformities.

The Philippines presented with the most severe mean CL/P, although its severity was not significantly larger when compared with other countries in Asia, excluding Laos. The 4 countries that demonstrated the most severe CL/P were all located in the Asia region. These countries were the Philippines, Vietnam, China, and Cambodia. More broadly, Asia was the region that presented with the greatest mean CL/P severity, a statistically significant difference compared with the other regions (*P* < .001). When excluding Laos, the entire Asia region was homogenous in its severity, whereby the mean angles were not statistically different between countries. It is unclear why the Laos subjects had significantly smaller columellar angles and Laos had the second smallest mean angle of any country.

The 2 other regions, Sub-Saharan Africa and the Middle East/Northern Africa, also showed homogeneity among their countries based on analysis-of-variance analysis. These results support the grouping of countries into the 3 regions that were chosen. The broad differences between regions highlight the presence of 1 or multiple underlying etiologies for CL/P that is more prominent in different areas of the world.

Our findings that the Asia region presents with the most severe cleft deformity correlate with other studies that identify Asian region as presenting with the highest incidence of cleft lips. Murray et al^5^ identified the birth prevalence of CL/P to be relatively high at 1.94 in 1000 births, and additional studies demonstrate that the Asian population has an elevated CL/P incidence as compared with white populations.^6^^-^8 Although there is no study connecting the risk factors identified for incidence with severity of CL/P, the apparent correlation between incidence and severity suggests that genetic and environmental factors that lead to differences in incidence may also contribute to the observed regional differences in CL/P severity.

One of the prominent debates in the field of cleft lip and palate is the deformity's cause. While there is considerable evidence on both sides of the nature versus nurture debate, the closest representation of risks is likely found in the middle. Approximately 70% of cases of CL/P occur as isolated entities with no other apparent cognitive or craniofacial structural abnormalities. Whereas twin studies and familial clustering studies have provided compelling evidence for a genetic component to nonsyndromic CL/P,^9^ few pedigrees show clear-cut Mendelian inheritance and most cases appear to be sporadic.^10^ Experimentation with animal models has identified mutations in interferon-regulating factor 6 (IRF6) and ventral anterior homeobox 1 (VAX1) as direct players in the development of nonsyndromic CL/P. First described in Van Der Wood syndrome, the role of IRF6 has been identified as a key determinate in keratinocyte proliferation and oral periderm formation to ensure proper palatal adhesion.^11^^-^13 Recent research has shown that IRF6 mutant mice exhibit a hyperproliferative epidermis that fails to undergo terminal differentiation and results in multiple epithelial adhesions occluding the oral cavity. Overexpression and variation of the *VAX1*, a gene known to encode a transcription regulator in craniofacial structures, has also been identified widely in the development of CL/P.^14^

As craniofacial malformations arise early in embryological development with modest recurrence rates, it has proven difficult to identify specific environmental factors associated with CL/P.^15^ Maternal smoking during the periconceptual period has been hypothesized to influence markers in the glutathione S-transferase 1 pathway and lead to deficiencies in detoxification.^16^^-^20 Folate deficiency has been suggested to influence risk of CL/P in both observational studies and interventional trials using supplementation.^21^ However, recent studies of folate receptor antibodies did not find an association with CL/P.^22^ Presently, a multifactorial model of inheritance is favored in which genetic risk factors of small, individual impact may interact with environmental covariates.^23^

Although our study appears to be the first comparing cleft lip severity among global communities, it is only one of many that scientifically analyzes CL/P severity. Al-Omari et al^24^ reviewed the prior approaches for assessing the degree of CL/P deformity and cited 5 studies that used clinical analysis, 23 studies that used 2-dimensional analysis (ie, photographs), and 8 studies that used 3-dimensional analysis (ie, computer-assisted tomography). However, in spite of the thorough consideration given to assessing CL/P severity, there remains active debate about the most effective method. Therefore, a major consideration for this study was determining the most effective method to compare CL/P in each region.

The ability to judge CL/P severity using photographs is based on the facial asymmetry that progresses with increasing CL/P severity. However, photographs convey a lot of complex information, which is difficult to standardize for objective analysis. Studies that have investigated whether photographs can be used to accurately characterize the level of cleft deformity utilize 2 general methods of analysis. The first approach utilizes anthropometric measurements that provide objective quantitative information about the deformity. However, each measure does not equally reflect the overall appearance of the deformity, and therefore the significance of each measure is not standardized.^4^^,^^25^^-^29 The second approach is to utilize the subjective opinion of experienced medical professionals to provide comparative rankings of severity for a group of subjects.^30^^-^37 While this method allows for analysis of the general appearance of the deformity, the reliance on subjective opinion excludes reliable comparisons across studies and allows for multiple confounding variables. Furthermore, although some studies were able to establish consistency among multiple surgeons, the use of this method is not feasible in large-scale studies, such as this one, which examines 760 photographs.

Fisher et al^3^ published a study that analyzed the correlation of various anthropometric measurements to the severity ranking as assessed by multiple experienced plastic surgeons. The result of the study effectively used both of the aforementioned methods and identified 2 objective measurements that best correlated with the assessment of CL/P severity according to surgeons. Our study used the Fisher paper findings, which identified the columellar angle as the most informative anthropometric measurement, to analyze the photographs taken during Operation Smile's WJS. It appears that this method effectively minimizes the limitations of both anthropometric and subjective analysis, thereby providing an accurate and standardized measure of CL/P based on medical photographs.

Assessing the CL/P deformity through medical photographs is the most common approach to objectively measure severity.^24^ The reliability of photographic data has improved because of the emphasis made by earlier studies to standardize the view of CL/P deformities.^24^ The frontal basal view is generally cited as the ideal position for photographs of CL/P, and all of the medical photographs for this study were standardized basal view images.

## AREAS FOR IMPROVEMENT

While considerable attention was given to determining the ideal method for assessing severity, other aspects of the study require further investigation to remove possible sources of error. Our study did not balance the different regions studied on the basis of the ages of subjects. While the vast majority of patients included in the analysis were infants or toddlers, multiple subjects appeared to be adolescent or middle age. However, one strength of our study was ensuring that there was no statistically significant difference in gender ratio within regions, which is important when considering the gender differences in CL/P severity.

Another variable that our study did not equilibrate was the number of subjects within each of the countries and regions that were compared. As a result, it is likely that some of the countries had a larger standard deviation than other countries, not because of intrinsic differences in the population, but rather because of differences in the number of subjects analyzed. For example, Jordan had the fewest subjects at 17, which was nearly 10 times fewer than Ethiopia, which had the most subjects at 166. However, by combining multiple countries into a more inclusive region, the disparity between individual countries was minimized. For example, Ethiopia and Jordan were included into the same region of North African-Middle East, thereby offsetting these outliers. Nonetheless, the Middle East-North Africa region had a total of 112 subjects, which was significantly less than the 352 and 316 of Asia and Sub-Sahara Africa, respectively. The discrepancy may have led to the large standard deviation seen in the Middle East region, which likely contributed to the inability to establish a statistically significant difference with Africa. Overall, the study still compares relatively large subject populations, and while more subjects are always welcomed, the large number of subjects in this study contributes to the significance of the findings.

## CONCLUSION

The results of this study suggest a heterogeneous pattern of global cleft lip severity. The severity was most severe in the East Asia region compared with other regions of the world, with the Philippines demonstrating the most severe mean CL/P deformity. The pattern of CL/P severity appears to mirror the pattern of CL/P incidence, suggesting that genetic and environmental factors contribute to both aspects of this deformity.

## Figures and Tables

**Figure 1:**
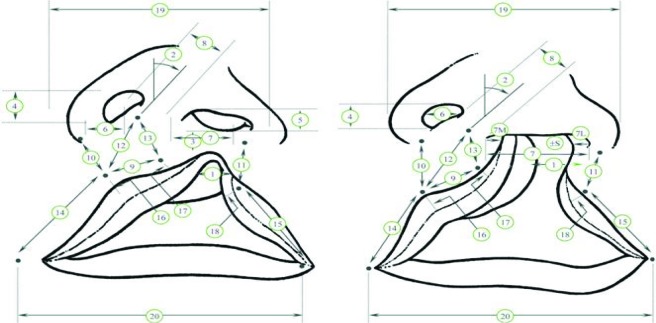
Measurements for left complete and incomplete unilateral cleft lip. Used with permission from Fisher et al.^3^

**Table 1:** A total of 760 patients with primary unilateral cleft lips treated by Operation Smile during November 2007^*a*^

						Interval for Mean	
Region	Country	N	Mean	SD	SE	Lower Bound	Upper Bound
Middle East/North Africa							
	Egypt	39	25.38	11.32	1.813	21.72	29.05
	Morocco	56	30.18	13.176	1.761	26.65	33.71
	Jordan	17	33.24	12.24	2.969	26.94	39.53
Sub-Sarahan Africa							
	Ethiopia	166	31.66	13.533	1.05	29.58	33.73
	Kenya	81	32.72	11.403	1.267	30.19	35.24
	Madagascar	69	35.43	17.735	2.135	31.17	39.7
East Asia							
	Philippines	144	40	15.237	1.27	37.49	42.51
	Vietnam	94	37.02	14.744	1.521	34	40.04
	China	34	36.32	16.574	2.842	30.54	42.11
	Cambodia	35	36	14.593	2.467	30.99	41.01
	Laos	45	29.11	15.458	2.304	24.47	33.76

^*a*^ The study analyzed photographs of 351 patients from Asia (China, Philippines, Vietnam, Laos, and Cambodia), 112 patients from the Middle East and North Africa (Jordan, Egypt, and Morocco), and 297 patients from Sub-Saharan Africa (Ethiopia, Kenya, and Madagascar).
